# A Genome-Wide Collection of *Mos1* Transposon Insertion Mutants for the *C. elegans* Research Community

**DOI:** 10.1371/journal.pone.0030482

**Published:** 2012-02-08

**Authors:** Elodie Vallin, Joseph Gallagher, Laure Granger, Edwige Martin, Jérôme Belougne, Julien Maurizio, Yohann Duverger, Sarah Scaglione, Caroline Borrel, Elisabeth Cortier, Karima Abouzid, Maité Carre-Pierrat, Kathrin Gieseler, Laurent Ségalat, Patricia E. Kuwabara, Jonathan J. Ewbank

**Affiliations:** 1 Centre de Génétique et de Physiologie Moléculaires et Cellulaires, CNRS UMR 5534, Campus de la Doua, Villeurbanne, France; 2 Université Claude Bernard Lyon 1, Villeurbanne, France; 3 School of Biochemistry, University of Bristol, Bristol, United Kingdom; 4 Centre d'Immunologie de Marseille-Luminy, Aix-Marseille University, Marseille, France; 5 INSERM, U1104, Marseille, France; 6 CNRS, UMR7280, Marseille, France; 7 Plateforme “Biologie de Caenorhabditis elegans”, CNRS UMS3421, Campus de la Doua, Villeurbanne, France; University of Pennsylvannia, United States of America

## Abstract

Methods that use homologous recombination to engineer the genome of *C. elegans* commonly use strains carrying specific insertions of the heterologous transposon *Mos1*. A large collection of known *Mos1* insertion alleles would therefore be of general interest to the *C. elegans* research community. We describe here the optimization of a semi-automated methodology for the construction of a substantial collection of *Mos1* insertion mutant strains. At peak production, more than 5,000 strains were generated per month. These strains were then subject to molecular analysis, and more than 13,300 *Mos1* insertions characterized. In addition to targeting directly more than 4,700 genes, these alleles represent the potential starting point for the engineered deletion of essentially all *C. elegans* genes and the modification of more than 40% of them. This collection of mutants, generated under the auspices of the European NEMAGENETAG consortium, is publicly available and represents an important research resource.

## Introduction

The nematode worm *Caenorhabditis elegans* has long been a model of choice for many areas of biological research because of its powerful genetics. The worm continues to attract researchers thanks to the extensive community-generated resources that have been developed for genetic and functional genomic studies. Recent advances have now made it possible to engineer specific changes to the *C. elegans* genome through homologous recombination. Two techniques have become popular, MosTIC, for *Mos1* excision-induced transgene-instructed gene conversion, [1], and MosSCI, for *Mos1*-mediated single-copy insertion [2]. Both methods rely on the availability of *C. elegans* strains carrying integrated copies of the heterologous *Mos1* transposon at defined genomic addresses. Chromosomal breaks can then be generated at a single locus through the controlled excision of the *Mos1* transposon. These breaks can be repaired through homologous recombination, using specifically designed transgenic templates, with homology arms that match the genomic sequence on either side of the *Mos1* transposon insertion site. In the case of MosTIC, the repair template can be engineered to introduce a mutation at a specific locus [1]. This method also makes it possible to “knock-in” reporter or affinity purification tags [3], thereby circumventing possible artifacts arising from transgene overexpression or chromatin-based position effects on gene expression. A third technique, MosDEL, can be used to generate *Mos1*-mediated targeted deletions of up to 25 kb [4], allowing null alleles to be generated with relative ease.

A large collection of molecularly defined *Mos1* insertion alleles would therefore be an extremely useful addition to the *C. elegans* toolkit. Previously, we documented the feasibility of generating such a collection [5] and detailed the implementation of a semi-automated high-throughput method for mutant production and screening [6]. Here, we describe how three laboratories involved in the European NEMAGENETAG project [7] successfully produced and characterized a large collection of strains carrying *Mos1* insertions. Theoretically, the collection of *Mos1* insertion alleles obtained during this project is sufficient to permit essentially every gene in the *C. elegans* genome to be knocked-out and more than 40% of genes to be modified in a targeted fashion. We have also devised and implemented a simple web-interface (MosLocator) to help researchers identify potentially useful alleles. As the *Mos1* strains are publicly available, they will be of general utility to the growing community of *C. elegans* researchers.

## Results

### Generating a large collection of independent *Mos1* alleles

In a pilot-scale experiment, we demonstrated the feasibility of generating and molecularly characterizing a large number of *Mos1* mutant alleles [5]. We subsequently described how the production of clonal lines of *C. elegans* with independent *Mos1* transposon insertions could be semi-automated, leading the way to the creation of a genome-scale collection of *Mos1* mutants [6]. The procedure used a standard protocol [8], which was initiated by mating two strains of worms, EG1470 and EN547, each carrying an extrachromosomal transgenic array. EG1470 animals provided the *Mos1* substrate on a transgene, and could be recognized because they also expressed a pharyngeal GFP marker. EN547 animals carried a heat-shock inducible *Mos1* transposase transgene associated with expression of GFP in the coelomocytes. From the resulting cross-progeny, individual hermaphrodites carrying both transgenic arrays were manually identified on the basis of the expression of the two GFP markers. These animals were then subjected to a transient heat-shock in order to activate the expression of the transposase from one array, thus allowing the *Mos1* substrate present on the other transgenic array to be mobilized. In a certain proportion of oocyte nuclei, the *Mos1* substrate integrated into the genome. The F1 progeny of these animals were then sorted using the Union Biometrica COPAS Biosort on the basis of GFP marker expression. In order to maximize *Mos1* insertions, and to prevent any further *Mos1* transposition, only individual worms that retained the substrate array, associated with pharyngeal GFP expression, but not the transposase array, were retained.

The fourth generation progeny derived from these F1 worms were passed through the Biosort and single non-fluorescent offspring for each original F1 worm were isolated and individually placed in culture for an additional 2 generations. The progeny obtained from each of these individual worms were then tested with an automated PCR protocol for the presence of a chromosomally-integrated *Mos1* transposon [6].

As previously reported, the transposition frequency and the final rate of obtaining *Mos1* insertion mutants varied considerably from week to week [6]. We noted that the frequency of *Mos1* transposition declined relatively rapidly when doubly transgenic worms were maintained in culture over time (Figure S1A). Thus, in an effort to reduce this variability, we modified our strategy, and generated a large pool of early generation doubly transgenic worms that were cryogenically stored. We found that when a newly thawed batch of doubly transgenic worms was used for an interval limited to 5–8 weeks, this had a favorable impact on the efficiency with which *Mos1* mutant strains were recovered at the end of the procedure. Typically 20–25% of F6 worms were found to harbor at least one *Mos1* insertion (Figure S1B).

**Figure 1:**
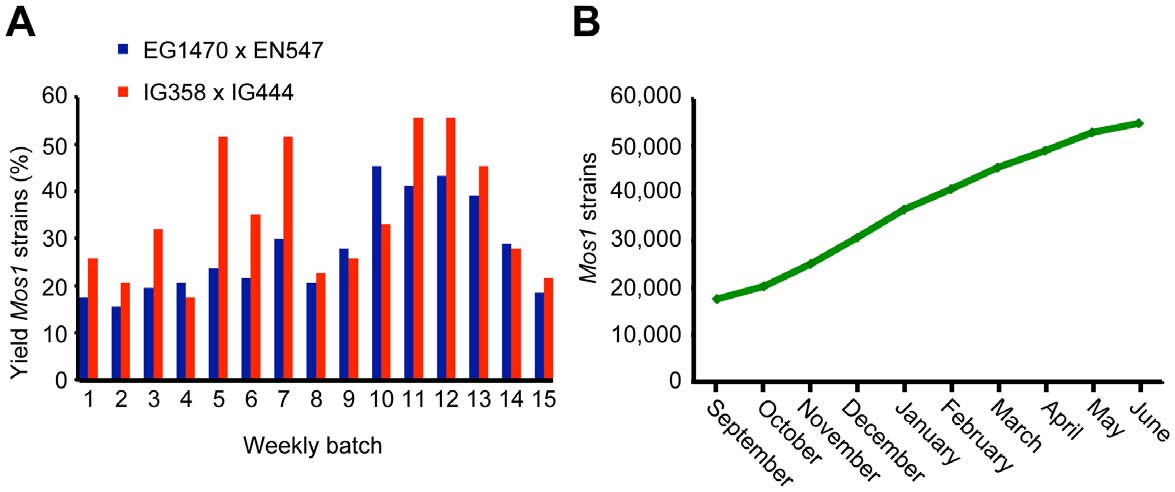
Production of the *Mos1* mutant collection. (A) A comparison of the efficiency of production of *Mos1* mutant alleles, generated with independent heat-shocks and measured with successive weekly batches of strains, starting with worms obtained through the mating of EG1470 and EN547 (blue bars), or IG358, an outcrossed strain derived from EG1470, with the new transgenic strain IG444 (red bars). (B) Graph showing the cumulative total of strains produced over the last 10 months of the project.

Despite this improvement, the manual sorting of large numbers of doubly transgenic worms was still laborious. We therefore produced a new strain (IG444) carrying a transgenic array composed of the *Mos1* transposase and the p*col-12*::dsRed marker construct [9], which is associated with bright red fluorescence in the epidermis. Introduction of this new fluorescent marker significantly improved the ease by which the doubly transgenic worms (Figure S1C) could be identified under a fluorescence binocular microscope. Given the decrease in *Mos1* mobilization that had been observed previously, and the problem of resident transposons in the starting strain described below, we mated IG444 with an outcrossed strain derived from EG1470, then established a large stock of doubly transgenic animals, which were passaged for a minimal number of generations before being frozen in multiple aliquots. We found that the strain showed a consistently high rate of *Mos1* transposition. When the two improvements were implemented, the yield of mutants increased markedly (Figure 1A) when compared to the overall 6.2% rate previously reported [6]. At the height of production, 5,927 strains were generated in a single month. By the end of the program, this procedure had been repeated over 150 cycles, and more than 300,000 lines of worms had been cultured over multiple generations and individually subjected to automated PCR-based analyses. As a result, more than 55,000 independent mutant strains were generated (Figure 1B).

Worms from each *Mos1*-containing strain were robotically cherry-picked from liquid culture in 96-well plates to the standard nematode solid medium in a 24-well format [6]. As a quality control step in the production process, a small aliquot of worms was removed from 28 randomly chosen wells in different plates (typically 24) and assayed by PCR for the presence of a *Mos1* insertion. In 100% of tests (n>300 wells), all strains yielded the expected *Mos1* amplicon. These plates were then sealed to prevent any cross-contamination between wells and sent on a weekly basis from Marseilles, France, to Lyon, France and to Bristol, UK. There they were molecularly characterized, in order to identify the site of insertion of the *Mos1* transposon in the *C. elegans* genome. A total of more than 55,000 clonal strains were dispatched, 46,473 strains were sent to Lyon and 8,615 strains to Bristol (Table 1).

**Table 1:** Summary of *Mos1* mutant production and characterization.

	Bristol	Lyon	Total
Strains sent	8615	46473	55088
Viable strains received	7967	46473	54440
Strains frozen	7967	25146	33113
Attempted molecular characterization	5280	46473	51753
Sequenced PCR amplicon	1401	25146	26547
Insertion site identified	1297	12037	13334

### Molecular characterization of *Mos1* alleles

When the weekly batches of 24-well plates arrived in Lyon and Bristol, they were processed in a systematic manner similar to that previously described [5]. An aliquot of the worms was clonally transferred to a 96-well plate, and DNA was released by lysis. The DNA obtained in this manner was then subjected to digestion with one of two alternative endonucleases, MboI or HaeIII. Digestion was followed by ligation and an inverse PCR reaction was performed using a pair of nested primers [10]. The resulting PCR amplicons were analyzed by agarose gel electrophoresis. When a unique band above a threshold size was obtained (see Material and Methods), it was purified and sequenced. When multiple bands were observed, generally the strongest one was purified, re-amplified and again purified before sequencing. For 34.2% of the strains it was possible to obtain a sequence using this method. For the remaining strains, a second attempt was made to generate a specific amplicon using the alternative enzyme, and sequences were thus obtained for an additional 19.9% of the strains. In total, more than 90,000 digestions and 300,000 PCR amplifications were carried out, leading to the generation of 26,547 PCR amplicons that were sequenced.

The sequence obtained for each PCR amplicon was compared by BLASTN to the *C. elegans* genome to identify the site of the *Mos1* insertion. The insertion site could be unambiguously identified for approximately 50% of the sequences. Overall, a *Mos1* insertion site could be identified in 13,334 independent lines, roughly a quarter of the 54,440 strains processed by the two sites (Table 1, Table S1). For reasons that remain unclear, we observed that the sequencing quality around the *Mos1*-genomic DNA junction was sometimes poor, which made it difficult to determine the insertion site of some alleles with precision (<+/−10 base pairs). Taking this into account, and eliminating redundant alleles (see below), we molecularly characterized 10,858 new independent mutant alleles (Table S1).

### Archiving of *Mos1* alleles

The archiving strategies differed between the two sites. Due to the higher number of strains that were handled in Lyon (Table 1), it was decided to characterize molecularly the strains before freezing. Only those strains yielding a unique or major PCR amplicon were conserved. After removing an aliquot from each well for the molecular study, the remaining worms were allowed to grow and reproduce in the 24-well plates until the food was exhausted. At this stage, which generally occurred around 3 weeks after reception, worms from wells that generated a unique or major PCR amplicon were cryopreserved. More than 25,000 strains were frozen in Lyon (Table 1). The Bristol team opted to freeze half of the worms in deep well 96 well plates soon after receipt, and to process the other half for DNA extraction and *Mos1* PCR amplification (Table 1).

### Analysis of the distribution of *Mos1* alleles across the genome

**Figure 2:**
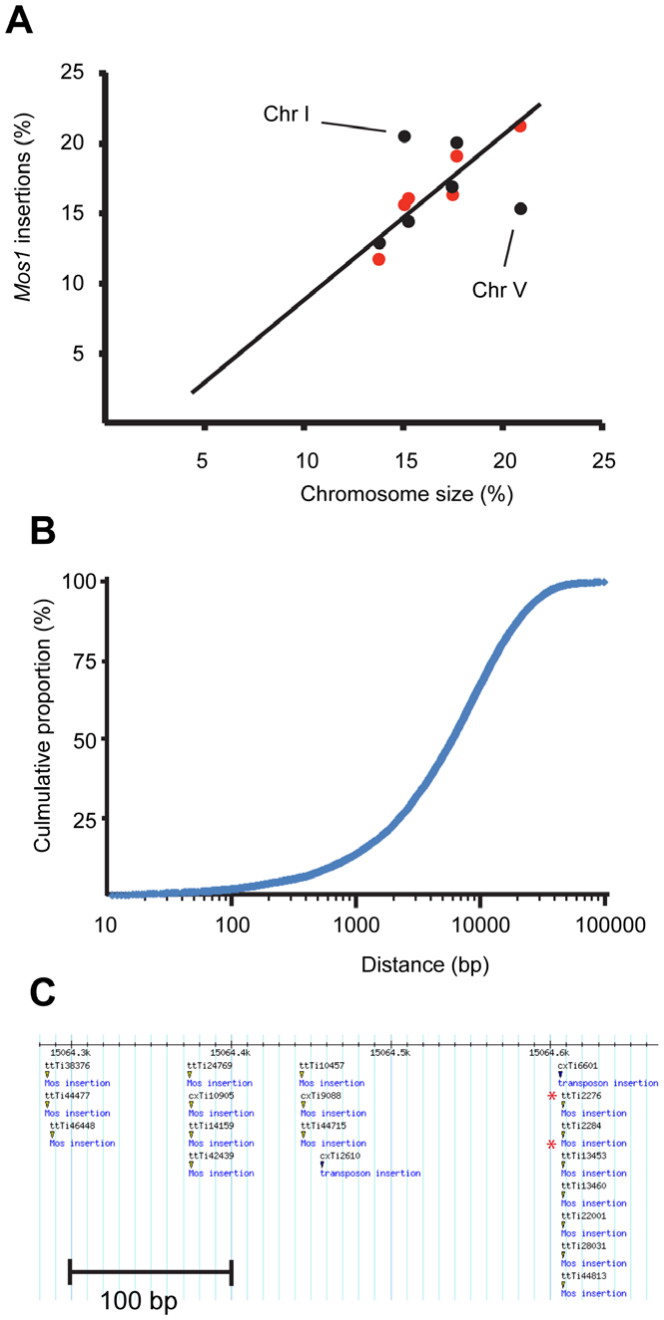
Distribution of *Mos1* alleles. (A) Graph showing the relationship between chromosome length (as a percentage of the whole nuclear genome) and the proportion of *Mos1* alleles per chromosome reported in a previous study [5], and the 10,858 alleles obtained in the current project (black and red circles, respectively). The outliers, concerning chromosomes I and V, from the previous study are highlighted with lines. (B) Distribution of distances from one *Mos1* allele to the next, in a 5′ to 3′ direction along each chromosome. The graph shows the cumulative percentage of alleles that are separated by less than the indicated distance. (C) Concentration of *Mos1* alleles at the extreme right end of chromosome I (length 15,072,423 bp). The separation of the allele numbers indicates that almost all the alleles were generated independently, except in two cases (ttTi2276 and ttTi2284; ttTi13453 and ttTi13460), highlighted by an asterisk. This region was also preferentially targeted during the previous study as reflected by the presence of several cxTi alleles.

**Figure 3:**
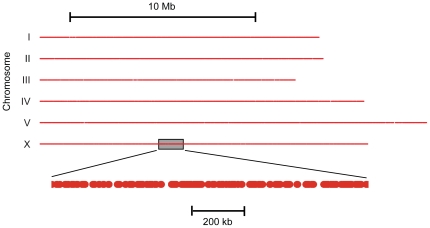
Genomic coverage of *Mos1*. Graphical representation of each *C. elegans* chromosome showing the regions of the genome that are potentially amenable to genome engineering using the publicly-available *Mos1* alleles; it is assumed that any point up to 1.5 kb away from a transposon-insertion site can be targeted. The bottom line is a magnified view of the boxed region on chromosome X.

Bioinformatics analyses were conducted to analyze the distribution of the complete collection of *Mos1* alleles and to evaluate its potential usefulness. As in some cases multiple redundant insertions were obtained, it was important to try to address whether this reflected a real bias of the *Mos1* transposon to insert at specific sites, or whether this was the consequence of experimental artifact. Of the 2,476 redundant alleles, 2,239 were found to have an insertion site that exactly matched that of an allele in the non-redundant set of 10,858 alleles (Table S1). When we looked at the allele numbers of such matching pairs, which reflects allele isolation history, we found that in almost 90% (1,972) of cases, the allele numbers differed by less than 25, indicating that they were derived from the same, or closest, 24-well plates. This strongly suggested, as discussed below, that the great majority of redundant alleles arose from experimental artifact. We therefore limited further analyses to the non-redundant set of 10,858 alleles. In our pilot study with 914 alleles, we reported a bias for insertions on chromosome I and against chromosome V [5]; with our new set of 10,858 alleles, this skewed distribution was not detected and the number of *Mos1* insertions found on each chromosome was proportional to the chromosome length (Figure 2A). The previously observed imbalance presumably represents a sampling bias. The average distance between neighboring alleles was 9,230 bp, with 33%, 67% and 95% of gaps being less than 3.2 kb, 10 kb and 30 kb, respectively. The single largest gap between adjacent alleles was less than 100 kb (Figure 2B, Table S1). There were, however, a few local “hot spots” for *Mos1* alleles. The extreme right end of chromosome III, and especially of chromosome I, for example, had a dense distribution (Figure 2C, results not shown). But otherwise, on the scale of each individual chromosome, the spread of *Mos1* alleles was relatively uniform (Figure 3).

Among the 10,858 new alleles spaced at least 10 bp apart, 6,345 *Mos1* insertions were found within 4,948 different genes, of which 4,586 were protein coding (Table S1). To test for any overall bias for insertion into intragenic or intergenic regions, we calculated the percentage of *Mos1* insertions contained within coding sequences (CDS) relative to the genome as a whole. Overall, 50.4% of insertions were in CDS (Table S1), which can be compared to the figure of 57.3% that we calculated to be the proportion of the genome that is in CDS. *Mos1* inserts at a TA dinucleotide [8]. There is a slight under-representation of TA (or AT) dinucleotides in CDS (55% of the genomic total by our calculation in WS220). Taken together, this suggests that there is no particular overall preference for insertion in intragenic or intergenic regions. The subset of genes that are expressed in the germ line, and so expected to be in an open chromatin context at the time when *Mos1* insertion occurs, might be predicted to be better targets. We used the overlap of 2 large-scale datasets [11], [12] to define a high-confidence list of 373 germline-expressed genes. Of these genes, 74 (19.8%) contained a *Mos1* insertion. As this is not significantly different (p>0.2, binomial test) from the number expected by chance, one can conclude that there was no enrichment for *Mos1* insertions in germline-expressed genes. We next evaluated the number of new *Mos1* insertions that fell into the coding regions of exons, which could provoke a loss of gene function. There are 1,816 such *Mos1* insertions, corresponding to 1,739 different genes. Again, these are distributed throughout the genome (Table S1). Overall, therefore, there appears to be little if any bias in the distribution of the *Mos1* insertions across the genome.

It has already been reported that the NEMAGENETAG collection allows essentially all *C. elegans* genes (99.4%) to be targeted by MosDEL [4]. The MosDEL method can effectively generate a deletion using a *Mos1* insertion located up to 25 kb away from the targeted gene. The MosTIC technique, on the other hand, permits genomic sites within 500 bp of a *Mos1* insertion to be modified efficiently, and can be used for targets up to 1.5 kb away [1]. We therefore calculated the number and proportion of protein-coding genes within 1.5 kb of a *Mos1* allele, including those generated in the pilot study (a total of 14,300). More than 40% of all protein-coding genes in the *C. elegans* genome (close to 8,500) are potential MosTIC targets (Table 2). It should, however, be pointed out that for many purposes, such as introducing a particular point mutation or inserting a sequence for a fluorescent protein or an affinity tag at the 5′ or 3′ end of a gene, researchers will want to target a specific region of a gene. So, the real utility of the collection requires evaluation on a case-by-case basis.

**Table 2:** Genome-wide distribution of *Mos1* mutant alleles.

Chr	Length (kb)	Number of protein coding genes	Number of *Mos1* alleles	Number of genes within 1.5 kb	% genes within 1.5 kb
I	15072 (15.0%)	2906 (14.2%)	2662 (18.6%)	1220 (14.4%)	42.0
II	15279 (15.2%)	3540 (17.3%)	2201 (15.4%)	1538 (18.1%)	43.4
III	13784 (13.8%)	2685 (13.2%)	1589 (11.1%)	1007 (11.9%)	37.5
IV	17494 (17.5%)	3321 (16.3%)	2445 (17.1%)	1302 (15.3%)	39.2
V	20920 (20.9%)	5134 (25.1%)	2851 (19.9%)	2141 (25.2%)	41.7
X	17719 (17.7%)	2828 (13.9%)	2552 (17.8%)	1276 (15.0%)	45.1
Total	100268	20414	14300	8484	41.6

Chr: chromosome.

### MosLocator: an online tool to aid *Mos1* allele identification

**Figure 4:**
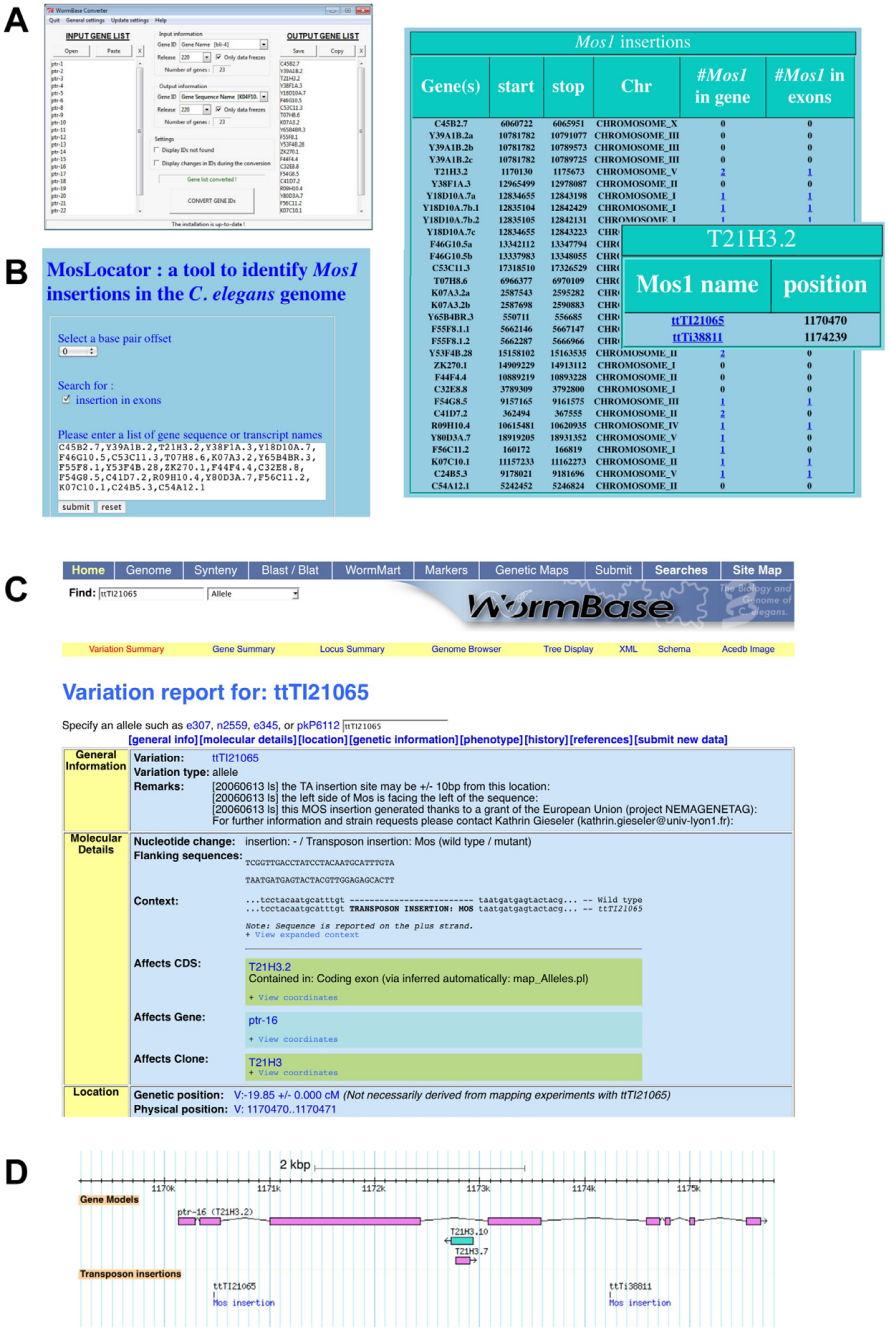
Finding *Mos1* alleles with MosLocator. (A) MosLocator (www.ciml.univ-mrs.fr/applications/MosLocator) finds *Mos1* alleles using gene sequence or transcript names. For large lists of genetic gene names, the gene sequence or transcript names can be obtained using WormMart, or here, using WormBase Converter (www.ciml.univ-mrs.fr/applications/WB_converter) [15]. In the example shown, the 23 *ptr* genes were used as input. (B) Screen grabs were captured to illustrate the use of MosLocator. Left panel: a list of sequence names was entered, and the search parameters were defined. Upper right panel: a display of the output for this search. Clicking on a non-zero number displayed in either of the last two columns, for example the “2” associated with the gene T21H3.2 (*ptr-16*), generates the display shown in the inset. This is a list of the 2 *Mos1* mutant alleles that are found within the gene T21H3.2. Each allele name is hyperlinked to Wormbase. (C) A partial view of the Variation report for the *Mos1* allele *ttTi21065* found on chromosome V at Wormbase (version WS225). (D) The genomic environment of the *ttTi21065* allele is displayed. The figure is a screen-grab from Wormbase.

The complete set of unique *Mos1* alleles has been entered into the community database Wormbase [13]. Those alleles that fall within exons or introns of annotated genes can be readily found on the corresponding gene page. On the other hand, any allele that falls into an intergenic region is not associated with a gene. Nonetheless, such alleles are potentially useful as the starting point for targeted genome engineering using MosTIC or MosDEL. Although all insertions can be seen using the Wormbase genome browser, or found with WormMart [14], these methods are somewhat cumbersome. This prompted us to devise a simple, but flexible tool called MosLocator that allows all the *Mos1* insertions within a set distance of a gene to be identified with ease (Figure 4). Because of the structure of the underlying database, MosLocator requires sequence or transcript names as an input. These identifiers can be found in Wormbase, or by using the online resource Wormbase Converter [15] that allows any of the common *C. elegans* identifiers to be converted to the gene sequence identifier (Figure 4A). Alleles associated with any number of genes can be found with MosLocator (Figure 4B). The tool also offers the possibility of returning only alleles that are found within or close to exons. When a *Mos1* transposon is found, the results window displays a direct link to the relevant page on WormBase (Figure 4C) that includes details of the allele and provides access to a graphical representation of its physical environment (Figure 4D). MosLocator is freely available at www.ciml.univ-mrs.fr/applications/MosLocator.

### Recovery of specific *Mos1* alleles from the collection

The 1,297 *Mos1* insertion alleles that were characterized in Bristol (numbered from *ttTi50009* and upwards) are identified as such in Wormbase and can be obtained by request to PK (email: p.kuwabara@bristol.ac.uk). The strains characterized in Lyon were originally maintained and distributed by the Segalat and Gieseler laboratories. They are now kept and distributed by a dedicated facility, “Biology of *C. elegans*” (UCBL CNRS UMS 3421). There are 12,942 strains available; 12,037 strains generated by the NEMAGENETAG consortium (*ttTi1* to *ttTi46473*) and 905 strains generated during the pilot study (*cxTi8901* to *cxTi10968*) [5]. Conditions of distribution are detailed at the website http://ums3421.univ-lyon1.fr. Briefly, researchers have the choice of asking for strains to be verified before being sent, or not. When verification is required, a specific pair of primers is synthesized that can be used to determine by PCR the presence of a particular *Mos1* insertion, and worms are sib-selected to derive a pure homozygous line [10]. This is reflected in a higher price and longer delay (currently, 250€ and up to 3 months, versus 35€ and 2–4 weeks for non-verified strains). At the time of writing, 792 *Mos1* strains had been requested from Lyon. A total of 451 strains (57% of requests) were sent without molecular verification. Among the other 341 requested strains, in 268 (79%) the *Mos1* element was found at the expected site, in 16 cases (5%) a viable strain was not recovered after thawing, and in 57 cases, the expected *Mos1* insertion was not found at the expected position.

We cannot exclude mistakes in labeling at the freezing and/or thawing steps, due to human error, as being a possible cause of not finding the expected *Mos1* insertion in more than 15% of cases in the Lyon collection. Another hypothesis, however, is that worms moved from one well where food was exhausted to another with food during the weeks that the 24-well plates were kept before worms were frozen.

To test this hypothesis, we randomly chose 15 “missing” insertions and thawed all the available strains that had been harvested and frozen from the 15 original 24-well plates. We then used our PCR approach to look for the specific “missing” *Mos1* insertions. In 14 out of 15 cases tested, the sought-after *Mos1* insertion was found in one or more strains of worms that came from the same original plate. As an extreme example, in addition to the expected *Mos1* insertion, a single well was also found to be PCR-positive for three independent *Mos1* insertions originally assigned to different wells on the same plate, while a single tested *Mos1* insertion was also detected in worms from as many as 11 wells of the same plate (data not shown). Although, as discussed below, this suggests that movement of worms from well to well was sometimes extensive, we would still expect to be able to recover more than 97% of requested *Mos1* insertions.

## Discussion

The collaboration of three European laboratories, in the context of the NEMAGENETAG consortium, has led to the generation of an extensive collection of individual *Mos1* alleles that should be of great use to the *C. elegans* research community. Because of the previously reported problems in recovering *Mos1* strains from frozen stocks, this project adopted a relatively laborious procedure involving the clonal culturing of strains for six generations with the aim of driving worms carrying *Mos1* insertions to homozygosity. The inverse PCR protocol does not allow one to distinguish between animals carrying homozygous or heterozygous insertions, but calculations suggest that only a small percentage of strains will not be homozygous [6]. Although requesting laboratories are asked to provide information on whether the strain they received was homozygous or heterozygous, the response has been sparse. We have, however, generated relevant data for a set of 200 strains, by genotyping 10 individual animals revived from a thawed stock, each associated with a particular *Mos1* insertion. With the caveat that this represents less than 2% of the total number of NEMAGENETAG *Mos1* insertion strains, and may not be representative, in 100% of cases an amplicon from the expected *Mos1* insertion was obtained in one or more of the tested animals. In only 53 cases (26.5%), however, were 10/10 tested animals positive for the expected *Mos1* insertion. That 10/10 *Mos1* positive worms were not always found may reflect the fact that the single worm PCR technique does not always work, leading to false negatives. Additionally, it is likely that the movement of worms between wells that we described above also contributed.

It is also important to note that only one *Mos1* insertion per strain was characterized, although additional insertions could be present, and in many cases more than one PCR amplicon was obtained after the first round of PCR amplification (unpublished results). At an intermediate point in the project, a further step was added, which involved sending all of the 24-well plates from Marseilles to the laboratory of J.L. Bessereau in Paris. Here, a small volume of buffer was added to each well to facilitate removal of an aliquot of worms in order to construct a pooled library (J.L. Bessereau, personal communication) before the plates were re-dispatched to their final destinations, Lyon and Bristol. This pooled library, Moslib, comprises more than 40,000 independent strains and will be the subject of a future publication. Results of an analysis of Moslib have indicated that each haploid genome contained on average 2.0 *Mos1* insertions (J.L. Bessereau, personal communication), which corresponds to roughly 80,000 independent insertions in the library since there is still every reason to believe that most frozen strains were homozygous for any *Mos1* insertions they might contain. This suggests that Moslib might contain up to 65,000 non-characterized insertions. Indeed, preliminary results suggest that a substantial number of previously uncharacterized insertions can be recovered from Moslib (J.L. Bessereau, personal communication). With advances in technology, these can be identified using methods based on next generation sequencing [16].

It can be seen from Table 1 that the two sites were roughly equivalent in their success in going from attempting molecular characterization of a strain to assigning a genomic position for an allele (24.6% and 25.9% for Bristol and Lyon, respectively). They attained this level in very different ways. While Lyon had substantially better success at the PCR amplification step than Bristol (54.1% and 26.5%, respectively), Bristol had a much better success rate at the sequencing step than Lyon (92.6% and 47.9%, respectively). This may reflect differences in reagent suppliers, or the competence of sub-contracted sequencing companies. As the disparities are large, it is unfortunate that direct side-by-side tests were not carried out to identify the important factors that influenced the results. With hindsight, one can only regret that overall, the project did not have the best of both steps, as combined this would have meant an overall 50% success rate, which would have potentially translated into a set of alleles twice the size of the current one.

Intronic *Mos1* insertions are unlikely to be mutagenic as they can be removed by splicing [8]. Furthermore, most strains are expected to be stable, and would not be expected to exhibit any gross developmental defects, as this would have been counter-selected during culture. It will be interesting to learn from requesting laboratories whether this is always the case, when an insertion is found within an exon of a gene previously associated with a visible phenotype.

Early in the project, multiple identical *Mos1* insertions were found in worms that were derived from supposedly independent lines. Thus, 280 of the 423 redundant alleles characterized in Bristol corresponded to just 2 insertion sites. The most frequent was also characterized repeatedly in Lyon; more than 350 alleles all with the same insertion site (I:12456295..12456296, +/− 10 bp) were found in total. Similarly, 239 and 26 insertions at the second site (IV:1136537..1136538, +/− 10 bp) were characterized in Bristol and Lyon, respectively. As multiple strains carrying the same allele were found at both laboratories, the problem must have arisen in Marseilles. This could have occurred if the double transgenic starting strains accumulated *Mos1* insertions at a low frequency even in the absence of forced expression of the recombinant transposase. Although this was not expected, and had not previously been reported (JL. Bessereau, personal communication), PCR analysis of non-transgenic progeny from the starting strain did reveal the presence of at least one resident *Mos1* insertion. To prevent the problem from reoccurring, the non-transgenic progeny from the starting strain were systematically checked for the absence of *Mos1* insertions before their transgenic siblings were subject to heat-shock. Once this technical problem had been overcome, any remaining minor redundancy was generally restricted to a single laboratory. Only 2 *Mos1* insertions sites out of the 1278 characterized redundantly in Lyon were also found in Bristol. As this laboratory-specific redundancy was also generally confined to single 24-well plates, it is likely to have arisen from cross-contamination during molecular characterization of the worms, such as might result if worms moved from one well to another. This is expected to occur more often when worms are starved, and food can be found in an adjacent well. Otherwise, as in any large-scale project, sample-handling errors will have also contributed to this small problem. While the production procedure in Marseilles was almost fully automated [6], the downstream molecular characterization, sequence analysis, and archiving involved intensive human intervention. Given the amplitude of the project, dealing with hundreds of thousands of samples, and the typical rate of human error, which experience suggests is in the order of several percent, future endeavors would clearly benefit from investment in the development of fully automated procedures. This is especially true as towards the end of the project, production capacity in Marseilles exceeded the capacity of the other partners.

In our analyses, we chose to be conservative, and only considered alleles more than 10 bp apart to be unique. The figures presented may therefore be a slight under-estimate of the number of unique strains obtained. Regardless, the generation of more than 10,000 characterized transposon alleles will undoubtedly be a boon to the *C. elegans* research community.

## Materials and Methods

### Mutagenesis and mutant isolation

*Mos1*-mediated mutagenesis was performed essentially as reported previously [6], except that during the later part of the project, worms with the genotype (*wt*; *frEx113*[pJL44(transposase);p*col-12*::DsRed]; *oxEx229*[Mos1,p*myo-2*:::GFP]), made by crossing IG444 (*wt*; *frEx113*[pJL44(transposase);p*col-12*::DsRed]) with IG358, a twice-outcrossed derivative of EG1470 (*wt*;*oxEx229*[Mos1,p*myo-2*:::GFP]), were used. Plates were sealed with AeraSeal cellular culture film (Excel Scientific, Victorville, CA, USA) before being dispatched from Marseilles.

### Inverse PCR and sequence analysis

An aliquot of each well from the 24-well plates was removed by washing with 10 µl of worm lysis buffer (10 mM Tris-HCl, pH 8.3; 50 mM KCl; 2.5 mM MgCl_2_; 0.5% Tween-20, and 100 µg ml^−1^ proteinase K) and transferred to wells in 96-well plates. Worm lysis was performed by freezing the plates at −80°C for at least 30 min, then incubating them for 60 min at 60°C, followed by 15 min at 95°C. Lysates were digested for 2 hours by one of the following enzymes: *Hae*III or *Mbo*I. The enzymes were then heat inactivated and the digested DNA ligated using standard protocols. Then 3 µl of each ligation reaction was used as substrate in a PCR reaction with primers oJL103-oJL114 [8]. A nested PCR was performed on 0.03 µl of the first reaction, using primers oJL115-oJL116 [8], [10]. PCR products were analyzed on a 1.8% agarose gel. When a unique band was seen, with a size greater than 270 bp for the *Mbo*I digestion protocol or 325 bp when using *Hae*III, the product was purified. When multiple bands were seen, the strongest one was re-amplified, applying the same size limits as above to ensure that only amplicons containing a *Mos1* insertion and flanking genomic DNA were sequenced. The sequence of each PCR product was compared to the *C. elegans* genome by BLASTN at the NCBI. Strains that gave PCR products for which the BLAST result was ambiguous, for example when the sequenced PCR fragment matched several genomic regions, were not characterized further.

### Cyropreservation

Starved worms were washed off wells with M9 and transferred to 1.8 ml cryovials (Nunc). Three vials were prepared from each well. They were frozen following standard protocols [17]. For permanent storage, one cryovial was kept in a −80°C freezer, and the other two were stored in liquid nitrogen. Alternatively, worms were mixed with freezing buffer and transferred to deep-well tubes in a 96-well format and frozen at −80°C in a Styrofoam container to slow the rate of freezing.

### Bioinformatic analyses and MosLocator

Because of changes in database structure at mining.wormbase.org, it was decided to develop a stand-alone tool to locate *Mos1* insertions relative to any given gene. A local MySQL database based on the publically available genome feature tables (from ftp://ftp.sanger.ac.uk/pub2/wormbase/releases/WS225/genomes/c_elegans/genome_feature_tables/GFF2/c_elegans.WS225.gff.gz) was established. The graphic interface was coded in PHP and Javascript. Submitted gene sequence or transcript names were sent as MySQL queries to the database to obtain genomic coordinates (entire gene and exons, if appropriate). These were then in turn compared to the genomic coordinates of the complete set of *Mos1* insertions present in a static file derived from WS225. The code is available on request.

To determine the potential genome coverage of the *Mos1* alleles for MosTIC, using WS225, an R script was written to concatenate any contiguous sequence within 1.5 kb up- or down-stream of each allele. This was used to produce the graphic representation in Figure 3. A second R script counted the number of genes that were contained within, or overlapped with the *Mos1*-associated contigs. These scripts and the list of contiguous regions are available on request.

## Supporting Information

Figure S1 **Optimizing the yield of** ***Mos1*** **-containing strains.** The percentage of F6 lines found to contain at least one *Mos1* insertion by PCR varied as a function of the interval between the thawing of the starting double transgenic strain and the mobilization of the *Mos1* transposon by heat shock. (A) A typical fluctuation for a batch of worms that was used for 8 consecutive weeks after thawing. (B) The results obtained with 3 successive batches (indicated by the different colors) that were used only between weeks 5 and 8 after thawing. (C) A doubly transgenic animal resulting from the cross of the strains IG358 and IG444; red and green fluorescence were visualized simultaneously.
(PDF)

Table S1 **NEMAGENETAG** ***Mos1*** **alleles.** The complete list of the 13,334 alleles characterized during the NEMAGENETAG project. The genomic position of each insertion and its Wormbase reference (Var ID) are derived from the frozen release WS220. A set of 10,858 non-redundant alleles that are at least 10 bp apart is highlighted in color: in yellow when the *Mos1* insertion is contained within a coding exon, and otherwise in green. For this non-redundant set, the number of genes that are directly targeted by each allele is given, together with a corresponding GeneID, derived from the frozen Wormbase release WS220, as well the distance to the neighboring allele (going in a 5′ to 3′ direction along each chromosome) and, in the last column an indication of whether an allele is contained within a gene. There are 940 cases where there is a lack of concordance between this last column and the columns “Genes hit” and “Targeted Gene Name”; this reflects differences in data structure between the data sources used. Where multiple alleles exist for a given genomic position, the choice of allele was entirely arbitrary, and similarly for the 277 alleles that target more than one gene, the choice of the single GeneID displayed was arbitrary.
(XLS)

## References

[1] Robert V, Bessereau JL (2007). Targeted engineering of the *Caenorhabditis elegans* genome following Mos1-triggered chromosomal breaks.. Embo J.

[2] Frokjaer-Jensen C, Davis MW, Hopkins CE, Newman BJ, Thummel JM (2008). Single-copy insertion of transgenes in *Caenorhabditis elegans*.. Nature genetics.

[3] Gendrel M, Rapti G, Richmond JE, Bessereau JL (2009). A secreted complement-control-related protein ensures acetylcholine receptor clustering.. Nature.

[4] Frokjaer-Jensen C, Davis MW, Hollopeter G, Taylor J, Harris TW (2010). Targeted gene deletions in *C. elegans* using transposon excision.. Nature methods.

[5] Granger L, Martin E, Segalat L (2004). Mos as a tool for genome-wide insertional mutagenesis in *Caenorhabditis elegans*: results of a pilot study.. Nucleic Acids Res.

[6] Duverger Y, Belougne J, Scaglione S, Brandli D, Beclin C (2007). A semi-automated high-throughput approach to the generation of transposon insertion mutants in the nematode *Caenorhabditis elegans*.. Nucleic Acids Res.

[7] Bazopoulou D, Tavernarakis N (2009). The NemaGENETAG initiative: large scale transposon insertion gene-tagging in *Caenorhabditis elegans*.. Genetica.

[8] Bessereau JL, Wright A, Williams DC, Schuske K, Davis MW (2001). Mobilization of a Drosophila transposon in the *Caenorhabditis elegans* germ line.. Nature.

[9] Pujol N, Cypowyj S, Ziegler K, Millet A, Astrain A (2008). Distinct innate immune responses to infection and wounding in the *C. elegans* epidermis.. Curr Biol.

[10] Boulin T, Bessereau JL (2007). *Mos1*-mediated insertional mutagenesis in *Caenorhabditis elegans*.. Nature protocols.

[11] Pauli F, Liu Y, Kim YA, Chen PJ, Kim SK (2006). Chromosomal clustering and GATA transcriptional regulation of intestine-expressed genes in *C. elegans*.. Development.

[12] Kim SK, Lund J, Kiraly M, Duke K, Jiang M (2001). A gene expression map for *Caenorhabditis elegans*.. Science.

[13] Harris TW, Antoshechkin I, Bieri T, Blasiar D, Chan J (2010). WormBase: a comprehensive resource for nematode research.. Nucleic Acids Res.

[14] Schwarz EM, Antoshechkin I, Bastiani C, Bieri T, Blasiar D (2006). WormBase: better software, richer content.. Nucleic Acids Res.

[15] Engelmann I, Griffon A, Tichit L, Montanana-Sanchis F, Wang G (2011). A comprehensive analysis of gene expression changes provoked by bacterial and fungal infection in *C. elegans*.. PloS one.

[16] Paruzynski A, Arens A, Gabriel R, Bartholomae CC, Scholz S (2010). Genome-wide high-throughput integrome analyses by nrLAM-PCR and next-generation sequencing.. Nature protocols.

[17] Stiernagle T (2006). Maintenance of *C. elegans*.. http://www.wormbook.org.

