# Supplementary figures and images for: A Genome-Wide Collection of Mos1 Transposon Insertion Mutants for the C. elegans Research Community
Source: PLoS One. 2012 Feb 8;7(2):e30482. doi: 10.1371/journal.pone.0030482 (PMC3275553; doi:10.1371/journal.pone.0030482)

Figure S1

A

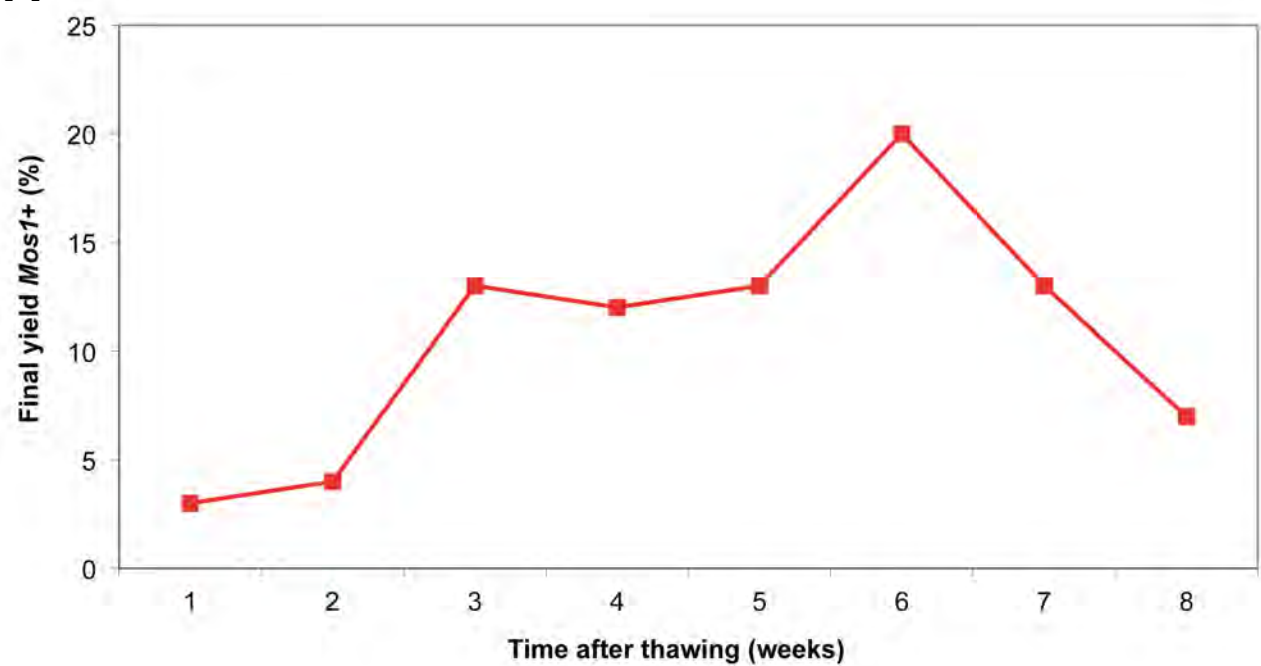

B

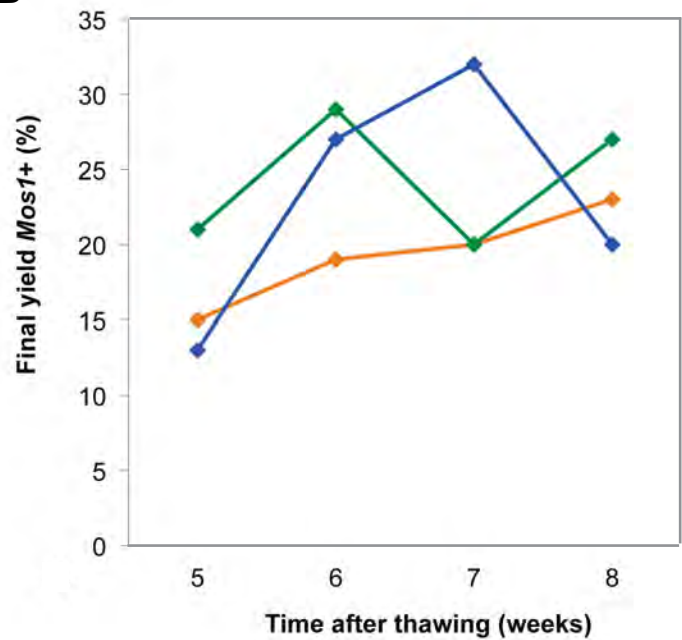

C

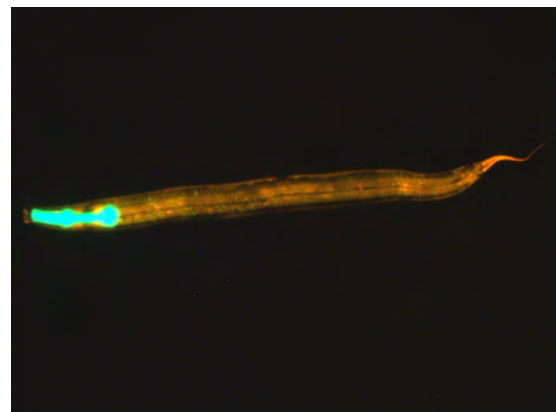

Supplement: Figure S1 — Optimizing the yield of Mos1 -containing strains. The percentage of F6 lines found to contain at least one Mos1 insertion by PCR varied as a function of the interval between the thawing of the starting double transgenic strain and the mobilization of the Mos1 transposon by heat shock. (A) A typical fluctuation for a batch of worms that was used for 8 consecutive weeks after thawing. (B) The results obtained with 3 successive batches (indicated by the different colors) that were used only between weeks 5 and 8 after thawing. (C) A doubly transgenic animal resulting from the cross of the strains IG358 and IG444; red and green fluorescence were visualized simultaneously. (PDF) [file pone.0030482.s001.pdf]
